# *Elateriospermum tapos* Yogurt Supplement in Maternal Obese Dams during Pregnancy Modulates the Body Composition of F1 Generation

**DOI:** 10.3390/nu15051258

**Published:** 2023-03-02

**Authors:** Ruth Naomi, Rusydatul Nabila Mahmad Rusli, Fezah Othman, Santhra Segaran Balan, Azrina Zainal Abidin, Hashim Embong, Soo Huat Teoh, Azmiza Syawani Jasni, Siti Hadizah Jumidil, Khaled Salem Yaslam Ba Matraf, Zainul Amiruddin Zakaria, Hasnah Bahari, Muhammad Dain Yazid

**Affiliations:** 1 Department of Human Anatomy, Faculty of Medicine and Health Sciences, Universiti Putra Malaysia, Serdang 43400, Malaysia; gs60018@student.upm.edu.my (R.N.); rusydatulnabila17@gmail.com (R.N.M.R.); hadizah_jumidil@upm.edu.my (S.H.J.); 2 Department of Biomedical Sciences, Faculty of Medicine and Health Sciences, Universiti Putra Malaysia, Serdang 43400, Malaysia; fezah@upm.edu.my (F.O.); khaled20111268@gmail.com (K.S.Y.B.M.); 3 Department of Diagnostic and Allied Health Sciences, Faculty of Health and Health Sciences, Management and Science University, Shah Alam 40100, Malaysia; santhra@msu.edu.my (S.S.B.); azrina@msu.edu.my (A.Z.A.); 4 Department of Emergency Medicine, Faculty of Medicine, Universiti Kebangsaan Malaysia, Kuala Lumpur 56000, Malaysia; hashimembong77@ukm.edu.my; 5 Advanced Medical and Dental Institute, Universiti Sains Malaysia, Penang 13200, Malaysia; soohuat@usm.my; 6 Department of Medical Microbiology & Parasitology, Faculty of Medicine & Health Science, Universiti Putra Malaysia, Serdang 43400, Malaysia; azmiza@upm.edu.my; 7 Borneo Research on Algesia, Inflammation and Neurodegeneration (BRAIN) Group, Faculty of Medicine and Health Sciences, Sabah Universiti Malaysia, Jalan UMS, Kota Kinabalu 88400, Malaysia; zaz@ums.edu.my; 8 Centre for Tissue Engineering and Regenerative Medicine, Faculty of Medicine, Universiti Kebangsaan Malaysia, Cheras, Kuala Lumpur 56000, Malaysia

**Keywords:** maternal nutrition, obesity, intergenerational effect, body composition, child

## Abstract

Maternal obesity is a key predictor of childhood obesity and a determining factor for a child’s body composition. Thus, any form of maternal nutrition during the gestational period plays a vital role in influencing the growth of the fetus. *Elateriospermum tapos* (*E. tapos*) yogurt has been found to comprise many bioactive compounds such as tannins, saponins, α-linolenic acid, and 5′-methoxy-bilobate with apocynoside I that could cross the placenta and exhibit an anti-obesity effect. As such, this study aimed to investigate the role of maternal *E. tapos* yogurt supplementation on offspring body composition. In this study, 48 female Sprague Dawley (SD) rats were induced with obesity using a high-fat diet (HFD) and were allowed to breed. Upon confirmation of pregnancy, treatment was initiated with *E. tapos* yogurt on the obese dams up to postnatal day 21. The weaning offspring were then designated into six groups according to their dam’s group (*n* = 8) as follows; normal food and saline (NS), HFD and saline (HS), HFD and yogurt (HY), HFD and 5 mg/kg of *E. tapos* yogurt (HYT5), HFD and 50 mg/kg of *E. tapos* yogurt (HYT50), and HFD and 500 mg/kg of *E. tapos* yogurt (HYT500). The body weight of the offspring was accessed every 3 days up to PND 21. All the offspring were euthanized on PND 21 for tissue harvesting and blood sample collection. The results showed that both male and female offspring of obese dams treated with *E. tapos* yogurt showed growth patterns similar to NS and reduced levels of triglycerides (TG), cholesterol, LDL, non-HDL, and leptin. Liver enzymes such as ALT, ALP, AST, GGT, and globulin, and renal markers such as sodium, potassium, chloride, urea, and creatinine levels significantly reduced (*p* < 0.05) in the offspring of *E. tapos* yogurt-treated obese dams with the normal histological architecture of the liver, kidney, colon, RpWAT, and visceral tissue that is comparable to NS. In toto, *E. tapos* yogurt supplementation of obese dams exerted an anti-obesity effect by preventing intergenerational obesity by reversing HFD-induced damage in the fat tissue of the offspring.

## 1. Introduction

Maternal obesity, also termed metaflammation [1], is a chronic low-grade inflammation that appears when the body mass index is more than 30 kg/m^2^ during pregnancy or the gestational period [2]. Being overweight or obese during the gestational period negatively impacts the metabolism of both the mother and the growing fetus. The child that is born to an obese mother will most likely develop obesity sooner or later [3]. Global data show that approximately 38.9 million obese pregnant subjects existed worldwide in the year 2014 [4], and it is expected that there will be at least 2.7 billion obese adults by the year 2025 [5]. According to a recent study published by Moschonis et al. in 2022, the prevalence of obesity has tripled over the last decade, and one in four children is now classified as obese [6]. Consumption of a high-fat diet (HFD) during the gestational period is one of the key factors for maternal obesity. In this, HFD intake remodels the placenta by inducing gut dysbiosis and stimulating the production of excessive oxidative stress in the placenta. In such conditions, lipoprotein lipase will be expressed in the placenta leading to a dysregulation in lipid metabolism. Thus, the offspring has a high possibility of inheriting obesity from its mother [7]. Thereby, maternal obesity is considered to be a vicious intergenerational cycle [8]. Maternal overnutrition and the presence of excessive inflammatory markers are the main factors influencing a child’s adiposity level. As such, the quality of the maternal diet plays a pivotal role in the body composition of the offspring. In consideration of this situation, maternal obese subjects need to follow anti-inflammatory dietary patterns during the gestational period to prevent the emergence of childhood obesity by suppressing maternal inflammation via dietary optimization [9]. 

Recent discoveries have shown that the consumption of modern medicines could stimulate adverse effects such as colorectal bleeding, increased blood pressure, and headaches. Hence, plants are known to be the best natural alternative to cater to maternal obesity and metabolism dysregulation in maternal obese subjects. This is mainly due to the presence of natural bioactive compounds in plants and the absence of documented cases of toxicity [10]. In this, a local tropical plant known as *Elateriospermum tapos* (*E. tapos*) has been shown to reverse adipose tissue hypertrophy caused by HFD intake during the gestation period in maternal obese subjects. In addition, mothers who supplemented with the raw extract of *E. tapos* during pregnancy showed a gradual reduction in body weight and calorie intake, while the activity of lipoprotein lipase was highly suppressed. This effect has been witnessed not only in the mothers but also in the offspring [11], which proves that *E. tapos* extract prevents the transgenerational inheritance of obesity and the obese gene product known as leptin in the offspring [12]. Studies claim that the natural anti-obesity effect of *E. tapos* could be due to the presence of various phytochemical compounds such as α-glucosidase, α-amylase, and pancreas lipase inhibitor enzymes, which inhibit fat and carbohydrate absorption. Aside from this, some other known isolated compounds from *E. tapos* extract include β-carotene, phenols, and flavonoids, which may enhance weight reduction via multiple signaling mechanisms by preventing the absorption of triglycerides and promoting lipid hydrolysis [13]. Simultaneously, yogurt consumption during pregnancy was found to supply an appropriate level of nutrients for both the mother and the child. Current investigations show that the yogurt supplemented group consumes a better quality diet compared to the control group, and there is a strong positive correlation between yogurt intake and weight loss [14]. As a matter of fact, the first medicinal plant-integrated yogurt (*E. tapos* yogurt) has been proven to suppress weight gain in obese dams and has proven its safety efficacy through in vivo model investigations [15]. Hence, this study has been designed to study the effect of maternal supplementation with medical plant-integrated yogurt (*E. tapos* yogurt) on the body composition of the male and female offspring using Sprague Dawley (SD) rats. 

## 2. Materials and Methods

### 2.1. Collection and Identification of E. tapos Seeds

Fresh *E. tapos* seeds were obtained from the Forest Research Institute of Malaysia (FRIM), Jengka, Pahang, and were sent to the Herbarium Biodiversity Unit at Universiti Putra Malaysia, for verification under the voucher code UPM SK 3154/17. 

### 2.2. Extraction of E. tapos Seeds

Ethanol extraction of *E. tapos* seeds was achieved by soaking 500 g of *E. tapos* in 2 L of 95% lab-grade ethanol for 7 days at room temperature. On the 7th day, the filtrate was obtained and filtered under reduced pressure using rotary evaporation. The precipitate was then collected and added to maltodextrin powder in a ratio of 1:1 before being dried in the oven [16]. The dried-powder form of the *E. tapos* extract was collected and stored at −20 °C until used [17]. 

### 2.3. Formulation of E. tapos Yogurt 

To formulate *E. tapos* yogurt, 100 mL of full cream (Dutch Lady Purefarm UHT) was boiled using a microwave for 10 min and was allowed to cool at room temperature. Once the temperature dropped, the starter culture comprising *Lactobacillus delbrueckii subsp. Bulgaricus ATCC 11842,* and *Streptococcus thermophilus APC151*, purchased from New England Cheesemaking Supply, was mixed in the milk and incubated for 7–8 h in a yogurt maker (Pensonic PYM-700). The formed yogurt was then refrigerated overnight at 2–4 °C. The following day, *E. tapos* powder was added to the formulated yogurt in a ratio of 2 g to 100 mL [18]. 

### 2.4. High-Fat Diet Preparation 

A high-fat diet (HFD) was prepared using the protocol described elsewhere [15]. For this, 50% of normal rat pellets (Gold Coin Feedmills) was mixed with 20% of milk powder (Dutch lady), 24% of ghee (Crispo), and 6% of corn oil (Vecorn). All the mixed ingredients were baked in an oven at 60 °C for 60 min. The baked HFD was then cut into pieces and stored in a freezer at 2–4 °C before being fed to the rats [15]. 

### 2.5. Experimental Animals 

All experiments were conducted upon obtaining approval from the Institutional Animal Care and Use Committee (IACUC), Universiti Putra Malaysia, with an ethic code of UPM/IACUC/AUP-R025/2022. For this study, 48 female SD rats weighing between 150 and 200 g were used. All rats were acclimatized for one week at 22 ± 3 °C with proper regulation of 12/12 h light/dark and fed with normal rat food purchased from Gold Coin Feedmills (M) Sdn Bhd, Selangor, Malaysia [19]. 

### 2.6. Obesity Induction, Mating, Gestation, and Weaning

The rats were fed with the HFD for 16 weeks to induce obesity. Upon successful obesity induction, the rats were divided into positive and negative control groups, and then into groups of 3 different concentrations of *E. tapos* yogurt treatment. All rats were allowed to mate with male rats in a ratio of (2:1). Manual palpation and vaginal smears were performed early in the morning to detect pregnancy [12]. The first sperm detection was recorded at 0 post-coitum [20]. Treatment was started immediately upon confirmation of pregnancy and up to weaning [12]. All pups were adjusted to 8–12 per dam on PND 1 followed by the initiation of *E. tapos* treatment for the dams. The treatment groups were as follows (*n* = 8); normal food and saline (NS), HFD and saline (HS), HFD and yogurt (HY), HFD and 5 mg/kg of *E. tapos* yogurt (HYT5), HFD and 50 mg/kg of *E. tapos* yogurt (HYT50), and HFD and 500 mg/kg of *E. tapos* yogurt (HYT500). Treatment for the dams was terminated on PND 21. The body weight of the male and female pups was documented every 3 days from PND 1 to PND 21 [21].

### 2.7. Plasma Biochemistry

All rats fasted for 12 h prior to blood collection on PND 21. The rats were then euthanized on PND 21 using a carbon dioxide overdose. About 5 mL of blood was collected using a plain tube. The tube was then centrifuged at a 3500× *g* force for 15 min. The serum was then collected and used for renal and liver profile analysis. Renal markers consisting of sodium (Na), potassium (K), chloride (Cl^−^), urea, creatinine, and liver enzymes such as alkaline phosphatase (ALP), alanine aminotransferase (ALT), aspartate transaminase (AST), total protein, albumin, total bilirubin, globulin, gamma-glutamyl transferase, and the albumin–globulin ratio were analyzed using an Alere Cholestech LDX^®^ Analyzer (3230 Bethany Lane Suite 8, Ellicott City, MD 21042, USA), in which the serum was placed in the cassette well of the main body, and the test was run. The value that appeared on the screen was recorded [15]. 

### 2.8. Lipid Profile and Leptin Analysis

The plasma lipid profile consisting of total cholesterol (TC), triglycerides (TG), low-density lipoprotein (LDL), and high-density lipoprotein (HDL) was analyzed using a diagnostic reagent test kit (Roche, Germany). To ensure complete clotting, the blood in the sealed Vacutainer^®^ was placed at room temperature, and, using a refrigerated centrifuge tube, the samples were centrifuged at a 1500× *g* force for 30 min followed by immediate incubation in an ice bath to maintain a temperature between 2 and 4 °C. The samples’ values were then read (total cholesterol, HDL, LDL, triglycerides, and non-HDL) using a Hitachi Automatic Analyzer 902 (Tokyo, Japan) [22]. The plasma leptin concentration was analyzed using a rat leptin ELISA kit supplied by MyBioSource. For this protocol, the plasma was centrifuged at a 1000× *g* force for 20 min. The supernatant was collected, and 50 µL of the sample was added to the sample well, while 50 µL of the standard solution was added to six standard wells as prescribed, while the blank well was left empty. Then, approximately 100 μL of an HRP-conjugate reagent was added to all wells except for the blank well. The plates were then covered with a closure plate membrane and incubated at room temperature for 60 min followed by washing. All the wells were washed four times, and chromogen solution A and chromogen solution B were added to all wells separately. The samples were mixed using gentle shaking, and the plate was wrapped in aluminum foil before incubation at room temperature for 15 min. As a final step, 50 μL of a stop solution was added to all wells, and the absorbance was read using an ELISA reader at 450 nm. The sensitivity was maintained at 1.0 ng/mL while conducting the ELISA protocol as described in the supplier’s guidelines [23].

### 2.9. Gross Organ Weight and Histological Analysis

Upon euthanasia, the gross weight of the liver, kidney, colon, brown adipose tissue, retroperitoneal white adipose tissue (RpWAT), gonadal fat, and visceral fats were documented, and all organs were then preserved in 10% of formalin. Tissue processing was performed on the liver, kidney, colon, RpWAT, gonadal fat, and visceral fats, and microtomes were used for sectioning. Sectioning was maintained at a thickness between 4 and 6 μm. The paraffin was allowed to float in the water bath to remove wrinkles and folds before being embedded into the glass slides. The glass slides containing sections of tissue were stained using the hematoxylin and eosin (H&E) staining protocol described elsewhere. The slides were then observed under a microscope, and the histological sections were captured using a microscope [24]. All histological slides were verified by a certified pathologist from Universiti Putra Malaysia. 

### 2.10. Quantitative Analysis of Fat Cells

Quantitative analysis of RpWAT and visceral fat tissue was performed by measuring the diameter (µm) of fat cells using a light microscope with an inbuilt ruler (KF2; Carl Zeiss, Hamburg, Germany) [25]. 

### 2.11. Statistical Analysis

Statistical analysis was performed using SPSS 27.0. Results were expressed as mean ± standard error of the mean (SEM) for body weight, organ weight, leptin profile, and liver and renal enzymes, while a normality test was run for all data. One-way ANOVA and post hoc Tukey tests were used to analyze the significant difference among groups. A probability of *p* < 0.05 was defined as a statistically significant result.

## 3. Results

### 3.1. Changes in Body Weight in Male and Female Offspring from PND 1 to PND 21

**Figure 1:**
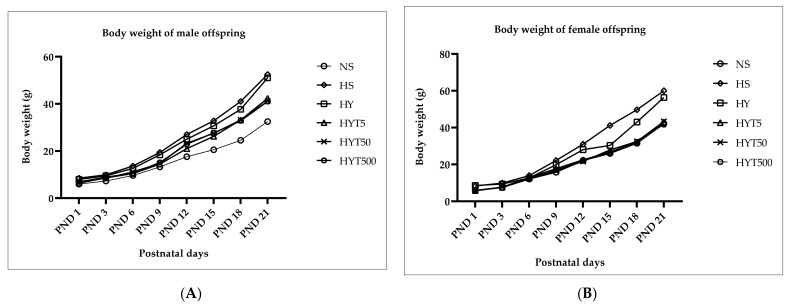
(**A**) The changes in body weight of male offspring from PND 1 to PND 21; (**B**) the changes in body weight of female offspring from PND 1 to PND 21.

Figure 1A,B shows the changes in body weight (BW) of the male and female offspring of *E. tapos* yogurt-supplemented obese dams from PND 1 to PND 21. The data in Figure 1A shows that the BW of the male offspring in the HS, HY, HYT5, HYT50, and HYT500 groups is significantly (*p* < 0.05) higher compared to the male offspring in the NS group on PND 1 up to PND 21. However, on PND 1, 3, and 6, the BW of the male offspring in the HYT5 group is significantly lower (*p* < 0.05) compared to the male offspring in the HS and HY groups, while there is no significant difference (*p* > 0.05) in BW of the male offspring in the HYT50 and HYT500 groups compared to the male offspring in the HS group. On PND 3, the BW of the male offspring in the HYT5, HYT50, and HYT500 groups is significantly lower (*p* < 0.05) compared to the male offspring in the HS group, while the mean value of the male offspring in the HYT500 group is similar to the male offspring in the HY group. On PND 6, the BW of the male offspring in the HYT5, HYT50, and HYT500 groups is significantly lower (*p* < 0.05) compared to the male offspring in the HS and HY groups. On PND 9, the BW of the male offspring in the HYT5, HYT50, and HYT500 groups is significantly lower (*p* < 0.05) compared to the male offspring in the HS group, and the mean values of the male offspring in the HYT5 and HYT50 groups are similar to the male offspring in the NS group. There is no significant difference (*p* > 0.05) in BW between the male offspring in the HYT5, HYT50, and HYT500 groups on PND 9, but the BW of the male offspring in the HYT500 group remains significantly higher (*p* < 0.05) than the male offspring in the NS group and significantly lower (*p* < 0.05) than the male offspring in the HS and HY groups. On PND 12 and 15, the BW of the male offspring in the HYT5, HYT50, and HYT500 groups is significantly lower (*p* < 0.05) compared to male offspring in the HS group and significantly higher (*p* < 0.05) compared to the male offspring in the NS group, while the BW of the male offspring in the HYT50 and HYT500 groups is significantly higher (*p* < 0.05) compared to the male offspring in the HYT5 group. On PND 18 and 21, the BW of the male offspring in the HYT5, HYT50, and HYT500 groups is significantly lower (*p* < 0.05) compared to the male offspring in the HS group and significantly higher (*p* < 0.05) compared to the male offspring in the NS group. However, there is no significant difference (*p* > 0.05) in BW between the male offspring in the HYT5, HYT50, and HYT500 groups on PND 21. The data from Figure 1B shows that the BW of the female offspring in the HS group is significantly higher (*p* < 0.05) compared to the female offspring in the NS group on PND 1 up to PND 21. A similar trend is observed in the female offspring in the HY group from PND 1, 3, 9, 12, 18, and 21; the BW of the female offspring in the HY group is significantly higher (*p* < 0.05) compared to the NS group, while no significant (*p* < 0.05) difference is observed between the HY and HS groups from PND 1, 3, 9, 12, 18, and 21. However, on PND 6 and 15, the BW of the female offspring is significantly lower (*p* < 0.05) compared to the HS group, and the mean value in the HY group is similar to the NS group on PND 6 and 15. Meanwhile, the BW of the female offspring in the HYT5, HYT50, and HYT500 groups is significantly lower (*p* < 0.05) compared to the female offspring in the HS and HY groups from PND 1 up to PND 21. In this, the data show no significant difference (*p* > 0.05) between the BW of the female offspring in the HYT5, HYT50, and HYT500 groups, and the mean values of the HYT5, HYT50, and HYT500 groups are similar to the BW of the female offspring in the NS group.

### 3.2. Gross Organ Weight of Male and Female Offspring on PND 21

Table 1 shows the changes in gross organ weight of the male and female offspring of dams supplemented with *E. tapos* yogurt on PND 21. As shown in Table 1, the gross organ weight of brown adipose tissue (BAT), liver, kidney, colon, and retroperitoneal white adipose tissue (RpWAT), and visceral and gonadal fat, including the length of the colon in male offspring, is significantly higher (*p* < 0.05) compared to the NS group. In male offspring in the HY group, the gross organ weight of the BAT, liver, kidney, and colon, and the length of the colon show no significant difference (*p* > 0.05) compared to the HS group. In this, the mean value of the male offspring’s liver is similar to the NS group as well. Meanwhile, the weight of RpWAT, visceral, and gonadal fat in the male offspring in the HY group is significantly lower (*p* < 0.05) compared to the HS group while significantly higher (*p* < 0.05) compared to the NS group. There is no significant difference (*p* > 0.05) for the HYT5, HYT50, and HYT500 groups in BAT, liver, kidney, colon, RpWAT, visceral, and gonadal fat, including the length of the colon compared to the NS group. Meanwhile, the weight of the colon, including colon length, and visceral and gonadal fat of the male offspring in the HYT5, HYT50, and HYT500 groups shows no significant difference (*p* > 0.05) compared to the HY group. The RpWAT in the male offspring in the HYT50 group shows no significant difference (*p* > 0.05) compared to the HY group. The weight of the liver in the male offspring in the HYT50 and HYT500 groups and of the colon in the HYT50 group shows no significant difference (*p* > 0.05) compared to the HS group. There is no significant difference (*p* > 0.05) between the male offspring in the HYT5, HYT50, and HYT500 groups in gross organ weight. For female offspring, the gross weight of the BAT, liver, kidney, RpWAT, visceral, and gonadal fat, including the length of the colon, is significantly higher (*p* < 0.05) compared to the NS group, as shown in Table 1. There is no significant difference (*p* > 0.05) in the female offspring’s colon weight between the NS, HS, HY, HYT5, HYT50, and HYT500 groups. Meanwhile, the organ weight of the BAT, liver, kidney, RpWAT, visceral, and gonadal fat in the HY group shows no significant difference (*p* > 0.05) in comparison with the HS group, while the length of the colon is significantly lower (*p* < 0.05) compared to the HS group. With the exception of a similar mean value for visceral fat in the HY and NS groups, all organs such as the BAT liver, kidney, colon, RpWAT, and gonadal fat in the HY group were significantly heavier (*p* < 0.05) compared to the NS group. The organ weight of the BAT, liver, kidney, RpWAT, visceral, and gonadal fat, including the length of the colon, is significantly lower (*p* < 0.05) in the HYT5, HYT50, and HYT500 groups of female offspring compared to the HS group, but there is no significant difference (*p* > 0.05) compared to the NS group. However, there is also no significant difference (*p* > 0.05) in the female offspring’s liver weight in the HYT500 group or the kidney weight in the HYT50 group in comparison with the HY group. There is no significant difference (*p* > 0.05) between the female offspring in the HYT5, HYT50, and HYT500 groups in gross organ weight.

### 3.3. Liver Profile of Male and Female Offspring on PND 21

Table 2 shows the liver profiles of the male and female offspring on PND 21. As shown in Table 2, there is no significant difference (*p* > 0.05) between the NS, HS, HY, HYT5, HYT50, and HYT500 groups in the total protein content of the male offspring. The plasma content of the globulin–albumin ratio, ALP, AST, ALT, and GGT is significantly higher (*p* < 0.05) in the male offspring in the HS group compared to the NS group, while there is no significant difference (*p* > 0.05) in plasma albumin content between the HS and NS groups. Meanwhile, the plasma concentration of albumin and bilirubin is significantly lower (*p* < 0.05) in the male offspring in the HS group compared to the NS group. In the male offspring in the HY group, there is no significant difference (*p* > 0.05) in plasma albumin, globulin, ALT, GGT, and bilirubin compared to the HS group. The mean value of the male offspring for albumin, globulin, the albumin–globulin ratio, and AST shows no significant difference (*p* > 0.05) compared to the NS group. However, the plasma content of ALP for the male offspring is significantly higher (*p* < 0.05) compared to the NS group, while significantly lower (*p* < 0.05) compared to the HS group. In the male offspring in the HYT5, HYT50, and HYT500 groups, the plasma content of albumin, the albumin–globulin ratio, AST, GGT, and bilirubin shows a significant reduction (*p* < 0.05) compared to the HS group, while no significant difference (*p* > 0.05) is observed compared to the NS group. The albumin content of the male offspring in the HYT50 group shows no significant difference (*p* > 0.05) compared to the HS group, while the HYT5 and HYT50 groups show no significant (*p* > 0.05) difference in plasma globulin content compared to the HS group. There is no significant difference (*p* > 0.05) between the HYT5, HYT50, and HYT500 groups for ALP content in male offspring; however, ALP is significantly lower (*p* < 0.05) compared to the HS group, while it is significantly higher (*p* < 0.05) compared to the NS, HYT50, and HYT500 groups. The mean value for ALP content in the male offspring in the HYT5 group is similar to the NS group, while the mean value for the HYT500 group is similar to the HY group. For ALT, the male offspring in the HYT5 group shows a similar mean value to the NS group, the HYT50 group shows a similar mean value to the HS group, while the HYT500 group shows a similar mean value to the HY group. There is no significant (*p* > 0.05) difference in plasma ALT content in the male offspring in the HYT5 and HYT500 groups. For the plasma concentration of GGT, the male offspring in the HYT5, HYT50, and HYT500 groups show no significant (*p* > 0.05) difference compared to the NS group. There is no significant (*p* > 0.05) difference in the male offspring in the HYT50 group compared to the HY and HYT500 groups compared to the HS and HY groups for plasma concentration of GGT. As shown in Table 2, there is no significant difference (*p* > 0.05) between the NS, HS, HY, HYT5, HYT50, and HYT500 groups in the total protein content of the female offspring. The plasma content of albumin and total bilirubin is significantly lower (*p* < 0.05) in the female offspring in the HS group compared to the NS group, while the levels of globulin, the albumin–globulin ratio, ALP, AST, ALT, and GGT are significantly higher (*p* < 0.05) in the female offspring in the HS group compared to the NS group. In the female offspring in the HY group, the levels of globulin, the albumin–globulin ratio, ALT, AST, ALP, GGT, and bilirubin show no significant difference (*p* > 0.05) compared to the HS group. However, the mean values of GGT and ALT for the female offspring in the HY group show no significant difference (*p* > 0.05) compared to the NS group. The mean value of albumin is significantly higher (*p* < 0.05) compared to the HS group while significantly lower (*p* < 0.05) compared to the NS group. Meanwhile, the level of the albumin–globulin ratio is significantly higher (*p* < 0.05) compared to the NS group. For all female offspring in the HY, HYT50, and HYT500 groups, the plasma concentration of albumin, globulin, the albumin–globulin ratio, ALT, AST, ALP, GGT, and total bilirubin shows no significant difference (*p* > 0.05) compared to the NS group. In this, the mean values of total bilirubin and globulin in the HYT5, HYT50, and HYT500 groups show no significant difference (*p* > 0.05) compared to the HS group. The mean values of albumin and the albumin–globulin ratio in the female offspring in the HYT50 and HYT500 groups is similar to the HY group, while the mean value of albumin in the female offspring in the HYT500 group shows no significant difference (*p* > 0.05) compared to the HS group. 

### 3.4. Renal Profiles of Male and Female Offspring on PND 21

Table 3 shows the renal profiles of the male and female offspring of *E. tapos* yogurt-supplemented obese dams on PND 21. As shown in Table 3, the level of sodium (Na) in the male offspring in the HS group is significantly lower (*p* < 0.05), while the levels of potassium (K) and creatinine are significantly higher (*p* < 0.05), compared to the NS group. Meanwhile, there is no significant difference (*p* > 0.05) in plasma chloride and urea concentration in the male offspring in the HS group compared to the NS group. In the male offspring in the HY group, the mean values of Na, K, chloride, and creatinine show no significant difference (*p* > 0.05) compared to the HS and NS groups. On the other hand, the plasma concentration of urea in the male offspring in the HY group is significantly lower (*p* < 0.05) compared to the HS group, with a mean value similar to the NS group. In the male offspring in the HYT5, HYT50, and HYT500 groups, the plasma concentration of Na is significantly higher (*p* < 0.05) compared to the HS group, and there is no significant difference (*p* > 0.05) compared to the NS group. In this, the male offspring in the HYT50 group shows no significant difference (*p* > 0.05) compared to the HY group. Regarding the plasma K concentration, the level is significantly lower (*p* < 0.05) in the male offspring in the HYT5 and HYT500 groups compared to the HS group, and there is no significant difference (*p* > 0.05) compared to the NS group. The plasma concentration of K in the male offspring in the HYT50 group shows no significant difference (*p* > 0.05) compared to the HS and NS groups. There is no significant difference (*p* > 0.05) in plasma chloride levels in the male offspring in the HYT5, HYT50, and HYT500 groups compared to the HS, HY, and NS groups. The plasma urea concentration shows no significant difference (*p* > 0.05) in the male offspring in the HYT5 and HYT50 groups compared to the HS and NS groups, while the urea level in the male offspring in the HYT500 group is significantly lower (*p* < 0.05) compared to the HS group and shows no significant difference (*p* > 0.05) between the HY and NS groups. The creatinine level in the male offspring in the HYT5, HYT50, and HYT500 groups is significantly lower (*p* < 0.05) compared to the HS group, with a similar mean value to the NS group. Similarly, the creatinine level in the male offspring in the HYT5 and HYT500 groups is significantly lower (*p* < 0.05) compared to the HY group, while there is no significant difference (*p* > 0.05) between the HYT50 and HY groups. As shown in Table 3, there is no significant difference (*p* > 0.05) between the NS, HY, HS, HYT5, HYT50, and HYT500 groups for plasma chloride and Na concentrations in the female offspring. The levels of urea and creatinine are significantly higher (*p* < 0.05) in the female offspring in the HS group compared to the NS group. In the female offspring in the HY group, the creatinine level shows no significant difference (*p* > 0.05) compared to the HS group, while the urea level shows a significant reduction (*p* < 0.05) compared to the HS group. In this, the creatinine level in the female offspring in the HYT5, HYT50, and HYT500 groups is significantly lower (*p* < 0.05) compared to the HS group, while the level of urea in the HYT5 and HYT500 groups is significantly lower (*p* < 0.05) compared to the HS group, with a similar mean value to the NS group. There is no significant difference (*p* > 0.05) between the HYT50 and HS groups in the female offspring’s urea concentration in plasma. The level of K is significantly higher (*p* < 0.05) in the female offspring in the HS group compared to the NS group, while the female offspring in the HY group show no significant difference (*p* > 0.05) in plasma K concentration compared to both the HS and NS groups. Meanwhile, the level of K is significantly reduced (*p* < 0.05) in the female offspring in the HYT5 and HYT50 groups compared to the HS group. The mean value of the female offspring in the HYT5 and HYT50 groups for K is similar to the NS group. There is no significant difference (*p* > 0.05) in plasma K concentration between the female offspring in the HYT500, HS, and NS groups.

### 3.5. Lipid Profiles of Male and Female Offspring on PND 21

Table 4 shows the lipid profiles of the male and female offspring of *E. tapos* yogurt-supplemented obese dams on PND 21. As shown in Table 4, the levels of total cholesterol, non-HDL, LDL, and triglycerides are significantly higher (*p* < 0.05) in the male offspring in the HS group compared to the NS group. The level of HDL is significantly reduced (*p* < 0.05) in the HS group compared to the NS group. However, the male offspring in the HYT5, HYT50, and HYT500 groups show a significant (*p* < 0.05) reduction in plasma total cholesterol, HDL, and triglycerides, and a significant increase (*p* < 0.05) of HDL, on PND 21 in comparison with the HS group; the mean value is similar to the NS group. For plasma LDL concentration, a significant reduction (*p* < 0.05) is observed in the male offspring in the HYT5 and HYT50 groups compared to the HS group. However, the plasma LDL level remains significantly higher (*p* < 0.05) in the male offspring in the HYT500 group compared to the NS group, with a similar mean value as the HS group. Thus, there is no significant difference (*p* > 0.05) between the male offspring in the HYT500 and HS groups for plasma LDL concentration. In this, the plasma levels of triglycerides, HDL, total cholesterol, non-HDL, and LDL show no significant difference (*p* > 0.05) between the male offspring in the HY group compared to the HS group and remains significantly higher (*p* < 0.05) compared to the NS group on PND 21. Meanwhile, as demonstrated in Table 4, the lipid profile of the female offspring of obese dams shows that the plasma concentrations of total cholesterol, non-HDL, LDL, and triglycerides are significantly higher (*p* < 0.05) in the HS group compared to the NS group. The level of HDL is significantly reduced (*p* < 0.05) in the HS group compared to the NS group. Meanwhile, the mean value for LDL in the female offspring in the HY group shows no significant difference (*p* > 0.05) in comparison with the HS group. However, the levels of total cholesterol, non-HDL, and triglycerides are significantly higher (*p* < 0.05) in the HY group compared to the NS group, but show no significant difference (*p* > 0.05) in comparison with the HS group. The mean value of HDL in the female offspring in the HY group is significantly higher (*p* < 0.05) compared to the HS group and shows no significant difference (*p* > 0.05) in comparison with the NS group. In the female offspring in the HYT5, HYT50, and HYT500 groups, the mean values for total cholesterol, HDL, non-HDL, LDL, and triglycerides for the HY group are significantly reduced (*p* < 0.05) compared to the HS group and show no significant difference (*p* > 0.05) in comparison with the NS group.

### 3.6. Leptin Levels in Male and Female Offspring on PND 21

**Figure 2:**
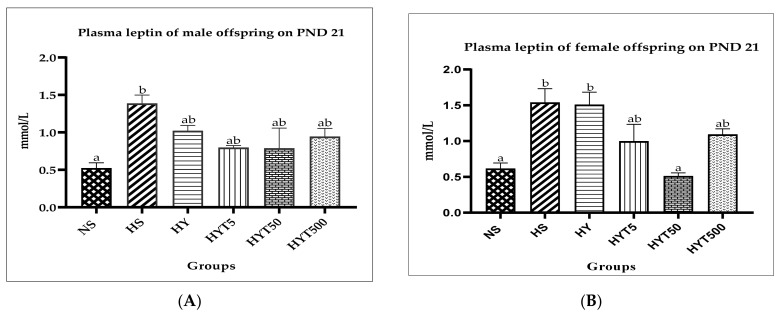
(**A**) Plasma leptin concentration of male offspring on PND 21; (**B**) plasma leptin concentration of female offspring on PND 21. NS: normal food and saline; HS: HFD and saline; HY: HFD and yogurt; HYT5: HFD and 5 mg/kg of *E. tapos* yogurt; HYT50: HFD and 50 mg/kg of *E. tapos* yogurt; HYT500: HFD and 500 mg/kg of *E. tapos* yogurt. Different letters indicate a significant difference at *p* < 0.05.

Figure 2A,B shows the plasma concentrations of leptin in the male and female offspring of *E. tapos* yogurt-supplemented obese dams on PND 21. As shown in Figure 2A, the plasma leptin concentration of the male offspring is significantly higher (*p* < 0.05) in the HS group compared to the NS group on PND 21. However, the male offspring in the HY group show a gradual decrease in plasma leptin concentration but show no significant difference (*p* > 0.05) compared to the HY group and show a similar mean value to the HY and NS groups. Meanwhile, the male offspring in the HYT5, HYT50, and HY500 groups show a gradual reduction in plasma leptin concentration but show no significant difference (*p* > 0.05) between the HS and HY groups; yet the mean value for plasma leptin concentration in the HYT5, HYT50, and HY500 groups is similar to the NS group. As shown in Figure 2B, the plasma leptin concentration of the female offspring is significantly higher (*p* < 0.05) in the HS group compared to the NS group on PND 21. Similarly, the plasma leptin concentration of the female offspring in the HY group is significantly higher (*p* < 0.05) in the HS group compared to the NS group on PND 21 but shows no significant (*p* > 0.05) difference compared to the HS group. Meanwhile, the plasma leptin concentration of the female offspring in the HYT5, HYT50, and HYT500 groups shows a gradual reduction compared to the HS group, while the mean plasma leptin concentration of the female offspring in the HYT50 group is significantly lower (*p* < 0.05) compared to the HS group and similar to the NS group. However, there is no significant (*p* > 0.05) difference in the plasma leptin concentration of the female offspring in the HYT5 and HYT500 groups compared to the HS group; yet the mean value of plasma leptin concentration of the female offspring in the HYT5 and HYT500 groups is similar to both the NS and HS groups. 

### 3.7. Histological Analysis of the Liver, Kidney, Colon, RpWAT, and Visceral Tissue of Male and Female Offspring on PND 21

**Figure 3:**
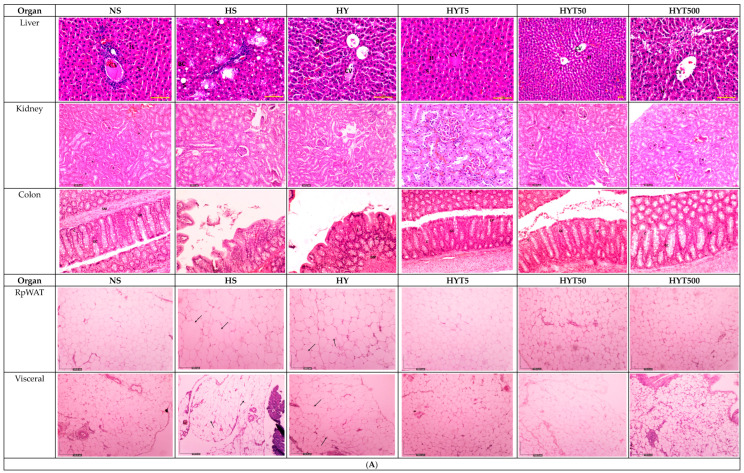

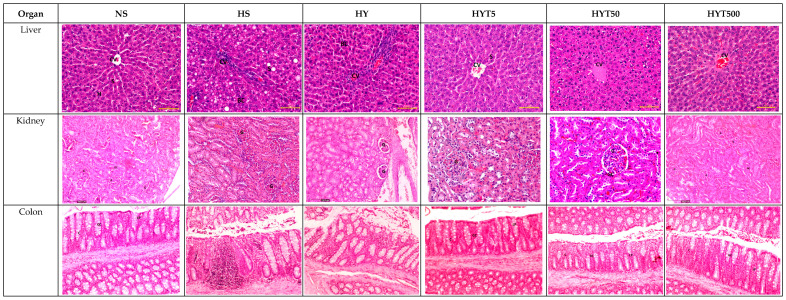

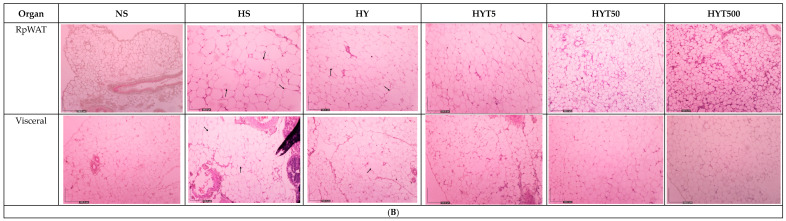
(**A**) The histopathological changes in liver, kidney, colon, RpWAT, and visceral fat tissue of male offspring of PND 21. HS shows abnormal strands of hepato-cytes (H), sinusoids (S), and central veins (CVs). Cell ballooning, steatosis, and >30% of lobular inflammation were seen. The liver histology in the HY group shows abnormal hepatocytes, sinusoids, and CVs with hepatocyte ballooning with lipid droplets. There is no presence of steatosis or lobular inflammation in the HY group. The liver histology for male offspring of the NS, HYT5, HYT50, and HYT500 groups shows a completely normal architecture. The kidneys of male offspring of the HS and HY groups show slight tubular dilation, the presence of abnormal lesions, and slight abnormalities of the renal corpuscle, while the liver histology for male offspring of the NS, HYT5, HYT50, and HYT500 groups shows a completely normal architecture. The colons in male offspring of the HS and HY groups show a detachment of epithelial cells, reduced mucosal content in the colonic wall, severe infiltration in the lamina propria, the presence of inflammation, and fat deposition in the muscle layer. The colon structure of the male offspring in the HYT5, HYT50, and HYT500 groups shows a completely normal architecture. There is severe fat hypertrophy in male offspring of the HS group in retroperitoneal white adipose tissue (RpWAT) and visceral fat tissue. (**B**) The histopathological changes in liver, kidney, colon, RpWAT, and visceral fat tissue of female offspring on PND 21. HS shows abnormal strands of hepatocytes (H), sinusoids (S), and central veins (CVs). Cell ballooning, steatosis, and >30% lobular inflammation were seen. The liver histology in the HY group shows abnormal hepatocytes, S, and CVs with hepatocyte ballooning with lipid droplets. There was no presence of steatosis or lobular inflammation in the HY group. The liver histology for female offspring of the NS, HYT5, HYT50, and HYT500 groups showed a completely normal architecture. The kidneys of female offspring in the HS and HY groups showed slight tubular dilation, the presence of abnormal lesions, and slight abnormalities of the renal corpuscle. The liver histology for female offspring in the NS, HYT5, HYT50, and HYT500 groups showed a completely normal architecture. The colons in female offspring in the HS and HY groups showed a detachment of epithelial cells, reduced mucosal content in the colonic wall, severe infiltration in the lamina propria, the presence of inflammation, and fat deposition in the muscle layer. The colon structure of the female offspring in the HYT5, HYT50, and HYT500 groups shows a completely normal architecture. Severe fat hypertrophy was present in female offspring of the HS group, being observed in retroperitoneal white adipose tissue (RpWAT) and visceral fat tissue. The arrow symbols refer to the fat hypertrophy.

Figure 3A,B shows the histological analysis of the liver, kidney, colon, RpWAT, and visceral tissue of male and female offspring of dams supplemented with *E. tapos* yogurt on PND 21. Similar histological features were observed in both the male and female offspring’s histological changes. As shown in Figure 3A,B, the male and female offspring in the HS group show abnormal strands of hepatocytes (H), sinusoids (S), and central veins (CVs). Cell ballooning, steatosis, and >30% of lobular inflammation were observed. Thus, for both the male and female offspring in the HS group, the liver scoring has been graded as 2. Similarly, the liver histology of the male and female offspring in the HY group shows abnormal hepatocytes (H), sinusoids (s), and central veins (CVs) with hepatocyte ballooning with lipid droplets. However, there is no presence of steatosis or lobular inflammation in the HY group. As such, for both the male and female offspring in the HS group, the liver scoring has been graded as 1. In this, the liver histology for the male and female offspring in the NS, HYT5, HYT50, and HYT500 groups shows a completely normal architecture. Thus, for the livers of both the male and female offspring in the NS, HYT5, HYT50, and HYT500 groups, the liver scoring has been graded as 0. Similarly, the kidneys of the male and female offspring in the HS and HY groups show slight tubular dilation, the presence of abnormal lesions, and slight abnormalities of the renal corpuscle, while the liver histology for the male and female offspring in the NS, HYT5, HYT50, and HYT500 groups shows a completely normal architecture. There is no difference in the histological structure of the kidneys of males and females in the HYT5, HYT50, and HYT500 groups compared to the NS group. Meanwhile, the colons of both the male and female offspring in the HS and HY groups show a de-attachment of epithelial cells, reduced mucosal content in the colonic wall, severe infiltration in the lamina propria, the presence of inflammation, and fat deposition in the muscle layer. However, the colon structure of the male and female offspring in the HYT5, HYT50, and HYT500 groups shows a completely normal architecture, which is comparable to the NS group. There is no difference in the histological structure of the colon of males and females in the HYT5, HYT50, and HYT500 groups compared to the NS group. There is severe fat hypertrophy in the male and female offspring in the HS group in retroperitoneal white adipose tissue (RpWAT) and visceral fat tissue. The degree of fat hypertrophy is less in the male and female offspring in the HY group in RpWAT and visceral tissue compared to the HS group. However, there is no adipocyte hypertrophy in the male and female offspring in the HYT5, HYT50, and HYT500 groups. There is no difference in the histological structure of RpWAT and visceral tissue of males and females in the HYT5, HYT50, and HYT500 groups compared to the NS group.

### 3.8. Quantitative Analysis of Fat Hypertrophy of Male and Female Offspring on PND 21

Table 5 shows a quantitative analysis of the fat hypertrophy of the RpWAT and visceral tissue of the female and male offspring on PND 21. The data show that the RpWAT and visceral tissue of the male and female offspring in the HS and HY groups is significantly heavier (*p* < 0.05) compared to the NS, HYT5, HYT50, and HYT500 groups. However, the RpWAT and visceral tissue of the male and female offspring in the HY group is significantly lower (*p* < 0.05) compared to the HS group. There is no significant difference (*p* > 0.05) in the quantitative analysis of the RpWAT and visceral tissue of the HYT5, HYT50, HYT500 groups compared to the NS group in either the male or female offspring on PND 21. 

## 4. Discussion

Maternal obesity is a vicious intergenerational cycle. Pre-pregnancy weight-gain greatly influences childhood obesity. This usually occurs through epigenetic modifications or inheritance of an obese gene, the melanocortin 4 receptor (MC4R). Being one of the key regulators for energy homeostasis, MC4R is the key predictor for body weight and food intake, and comprises the coding regions for leptin in offspring [26]. Practically, different growth patterns make it difficult to rule out obesity in children. However, a high level of adiposity in offspring is one of the vital indicators for developing obesity in later life [27]. Similarly, both male and female offspring of obese dams in the HS group showed significantly increased body weight from PND 1 to PND 21 compared to the NS group, proving the hypothesis of a vicious intergenerational cycle of maternal obesity. Similarly, dietary intake during pregnancy influences the body composition of offspring [28]. A recent toxicity study showed that *E. tapos* yogurt is safe to consume in amounts up to 2000 mg/kg/day [15]. Thus, this study was designed to study the effect of maternal *E. tapos* yogurt supplementation on the body composition of male and female offspring using Sprague Dawley (SD) rats. As shown in Figure 1A, the male offspring showed fluctuating changes in body weight in the HY5, HYT50, and HYT500 groups over time, yet remained lower compared to the HS group. This is relevant in regard to the previous claim of a gender-specific study that male offspring are more prone to developing obesity compared to female offspring [29]. In this, maternal prenatal supplementation plays a vital role in the weight gain of the child [30]. It was proven in this experimental study that supplementation with *E. tapos* yogurt during the gestational period influences the body composition of male and female offspring, in which the offspring of dams supplemented with *E. tapos* yogurt showed normal growth rates (body mass) regardless of varying concentrations (HYT5, HYT50, and HT500) and comparable to the offspring of the NS group. This is mainly due to the presence of bioactive compounds in the *E. tapos* yogurt. 

In addition, studies claim that maternal nutrition supplements during the gestational period will be transported through the umbilical cord to the growing fetus. In this regard, any compounds ranging between the molecular weights of 500 and 1000 g/mol can efficiently cross the placenta and reach the fetus easily, thereby exhibiting its effects [31]. Some of the identified bioactive compounds in *E. tapos* yogurt, such as 5′-methoxy-bilobate with a molecular weight of 582.5 g [32] and the lipophilic nature of apocynoside I [33], can cross the placenta and exhibit their anti-obesity effects on the growing fetus. This is because bilobetin is considered to be one of the strong inhibitors of pancreas lipase [34], which may result in a drastic reduction in fat absorption, eventually leading to weight loss [35]. Similarly, apocynoside I positively impacts the lipid profile of obese subjects by promoting weight loss [36]. Thus, this unrevealed and possible underlying cause of similar body mass in the male and female offspring in the HYT5, HYT50, and HYT500 groups is similar to the NS group. In addition, high-fat diet supplements during the gestational period tend to influence the mass of internal organs. The organ weight of the liver and kidney will increase by 5–7% in HFD-supplemented subjects due to fat tissue accumulation [37], while adipocytes hypertrophy can increase the mass of RpWAT, BAT, gonadal, and visceral fat tissue [38]. Similarly, prolonged HFD feeding could increase the crypt length of the colon, inflammatory infiltration [39], detachment of epithelial cells, and reduced mucosal content in the colonic wall [40,41]. Similar features as those described were observed in the organ weight as well as in the histological analysis of the liver, kidney, RpWAT, BAT, gonadal, and visceral fat tissue and the colon length of the male and female offspring in the HS group, further proving the establishment of the intergenerational obesity model in this study. However, these features were completely absent in the *E. tapos* yogurt-treated dams’ offspring in both organ weight and histological changes. One of the reasons could be due to the presence of tannins in *E. tapos* yogurt [15]. Tannins are known for their natural anti-inflammatory properties [42], and in the case of a low-grade condition such as obesity, tannins could potentially suppress inflammation by modulating the expression of cytokines and the formation of inflammatory molecules [43], particularly in adipocytes. Tannins have been proven to inhibit lipid accumulation, suppress the differentiation of adipocytes [44], and prevent the infiltration of fats into the liver [45]. Thence, the anti-inflammatory and antioxidant activity of tannins in *E. tapos* yogurt significantly explains the normal features and mass of the female and male offspring’s colon, kidney, liver, RpWAT, BAT, gonadal, and visceral fat tissue. 

Further, tannins are proven to increase the level of satiety and decrease the frequency of voluntary food intake. In reality, tannin exposure leads to an improvement in lipid profile [46]. An HFD load in the long term could result in increased plasma concentrations of TG, LDL, non-HDL, and total cholesterol [47]. Those characteristics have been observed in both genders of offspring of the HS and HY groups in this study, further proving the link between HFD and an alteration in lipid profile markers; yet those features seem to be absent in *E. tapos*-exposed groups (HYT5, HYT50, and HYT500). Again, the underlying reason could be due to tannins’ ability to interfere with the absorption of cholesterol, leading to a reduction of the cholesterol concentration in plasma [45]. Another study claims that the presence of tannins in fruit could express inhibitory activity against lipoprotein lipase, an enzyme that modulates the levels of HDL and LDL in plasma. In such conditions, hepatic lipase promotes the degradation of HDL cholesterol, causing the concentration level of HDL in plasma to rise [48]. This hypothesis is further supported by Gato et al. who, in 2012, witnessed similar data on the lipid profile of humans supplemented with tannin-rich fruit [49]. On the contrary, the plasma concentration of leptin in the placenta indicates the nutritional status of the child and is a marker for a high level of adiposity. A high level of leptin will stimulate neuropeptide Y (NPY), which will suppress activation of the hypothalamic–pituitary–adrenal (HPA) axis, causing the lipogenesis signaling mechanism to be stimulated [50]. Such a defect will be inherited from dams by their offspring, and is one of the primary reasons for familial causes of obesity. As a result, the offspring tend to develop resistance towards leptin and will possess an excessive level of leptin in their plasma [51]. This theory has been proven in this study, where the leptin concentration was significantly higher in the male and female offspring in the HS and HY groups. However, exposure to *E. tapos* yoghurt is able to reduce the plasma leptin concentration gradually, and the mean value was comparable to the offspring of the NS group. The presence of linolenic acids in *E. tapos* yogurt [15] could potentially stimulate lipolysis in the adipose tissue of the fetus [52], as the low plasma concentration of leptin in the offspring of dams treated with *E. tapos* yogurt evinces. 

In addition, a low Na^+^ plasma concentration correlates with fat mass and obesity. Preliminary studies show that obesity damages renal pressure natriuresis by stimulating the reabsorption of Na^+^ back into the kidneys. As a result, the plasma concentration of Na^+^ drops [53]. In this study, this phenomenon was observed in the male offspring of the HS group, while there was no effect on Na^+^ plasma concentration in the female offspring of the HS group. This might be due to the high body mass of the male offspring compared to the female offspring in this study. Meanwhile, a reduced level of serum potassium (K^+^) is associated with an increased level of serum TG, low HDL, and a large waist circumference. In short, metabolic component changes could induce electrolyte imbalance, and one of the common manifestations is reduced serum K^+^ [54]. In this study, both the male and female offspring of untreated obese dams showed a drastic reduction in K^+^ on PND 21, proving the speculation regarding an electrolyte imbalance in obesity. Electrolytes appear to be normally regulated in *E. tapos*-treated obese dams’ offspring, and this could be due to the presence of bioactive compounds such as saponins in *E. tapos* [12], as saponin can modulate Na^+^ and K^+^ levels by increasing the activity of Na^+^ and suppressing the activity of K^+^ [55]. Therefore, obesity increases the workload of the kidney, thereby stimulating the excessive release of tubular secretions, leading to a rise in plasma creatinine and urea [56]. This theory was proven in this study, as the male and female offspring of untreated obese dams possessed a high level of creatinine, but there was no effect on the serum urea level in the male offspring of obese dams on PND 21. This is again due to the ability of saponins to improve kidney function. According to the preliminary study performed by Kim et al., (2013), saponin removes excess levels of urea and creatinine from the body, thus decreasing their concentration in plasma [57]. Changes in liver enzymes are not an exception in the case of intergenerational obesity inheritance. Decreased levels of albumin in plasma have a positive correlation with body fat and inflammation in adipose tissue [58]. Other liver enzymes such as ALT, AST, ALP, and GGT were found to be increased in obese subjects. This could be due to the hepatic pathology arising due to HFD supplements and the presence of steatosis or hepatocyte ballooning due to lipid droplets [59]. Such pathological changes could induce oxidative stress, and the manifestation of decreased levels of serum bilirubin is common in obesity [60]. Laboratory assessments on the liver profiles of the male and female offspring of untreated obese dams (HS and HY) further support the hypothesis in this study. However, the presence of bioactive compounds in *E. tapos* yogurt is proven to reverse these pathological implications in offspring with HFD-induced obesity. Thus, all three different concentrations (HYT5, HYT50, and HYT500) of *E. tapos* yogurt-treated obese dams’ offspring showed comparable liver enzymes with NS offspring.

## 5. Conclusions

In toto, supplementation with *E. tapos* yogurt during the gestation period up to PND 21 of the obese dams resulted in significant changes in the body composition of their offspring regardless of gender. These include body weight, liver enzymes, renal markers, lipid profiles, leptin concentration, gross organ weight of fat tissues, and histopathological changes in vital organs. Thus, the outcome of this study gives preliminary data that the pre-treatment with *E. tapos* yogurt of obese dams during pregnancy could prevent the intergenerational transmission of obesity in the family, thereby proving the anti-obesity effects of *E. tapos* yogurt. Therefore, further study must be conducted to access the long-term effects of *E. tapos* yogurt on the transmission of the obesity gene. 

## Figures and Tables

**Table 1:** Gross organ weight of male and female offspring on PND 21.

Gross Organ Weight of Male Offspring on PND 21								
	BAT (g)	Liver (g)	Kidney (g)	Colon (g)	Colon Length (cm)	RpWAT (g)	Visceral (g)	Gonadal (g)
NS		4.97 ± 1.27 ^a^	0.92 ± 0.09 ^a^	0.75 ± 0.17 ^a^	11.50 ± 1.50 ^a^	0.25 ± 0.07 ^a^	0.30 ± 0.08 ^a^	0.29 ± 0.10 ^a^
HS	0.46 ± 0.02 ^b^	9.94 ± 1.15 ^b^	2.34 ± 0.13 ^b^	1.52 ± 0.09 ^b^	18.17 ± 0.83 ^b^	2.53 ± 0.34 ^b^	2.84 ± 0.37 ^b^	2.51 ± 0.42 ^b^
HY	0.48 ± 0.02 ^b^	7.66 ± 0.45 ^ab^	2.22 ± 0.13 ^b^	1.35 ± 0.05 ^bc^	15.80 ± 0.20 ^bc^	1.48 ± 0.01 ^c^	1.47 ± 0.04 ^c^	1.49 ± 0.15 ^c^
HYT5	0.30 ± 0.04 ^a^	5.72 ± 0.16 ^a^	1.09 ± 0.12 ^a^	0.82 ± 0.17 ^ac^	11.67 ± 0.67 ^ac^	0.58 ± 0.13 ^a^	0.73 ± 0.11 ^ac^	0.66 ± 0.15 ^ac^
HYT50	0.27 ± 0.02 ^a^	6.59 ± 0.18 ^ab^	1.22 ± 0.06 ^a^	0.99 ± 0.11 ^abc^	12.50 ± 0.50 ^ac^	0.76 ± 0.03 ^ac^	0.78 ± 0.02 ^ac^	0.81 ± 0.10 ^ac^
HYT500	0.31 ± 0.01 ^a^	6.19 ± 0.18 ^ab^	1.34 ± 0.03 ^a^	0.88 ± 0.08 ^ac^	11.83 ± 1.09 ^ac^	0.51 ± 0.09 ^a^	0.79 ± 0.09 ^ac^	0.82 ± 0.08 ^ac^
**Gross Organ Weight of Female Offspring on PND 21**								
	**BAT (g)**	**Liver (g)**	**Kidney (g)**	**Colon (g)**	**Colon Length (cm)**	**RpWAT (g)**	**Visceral (g)**	**Gonadal (g)**
NS	0.18 ± 0.04 ^a^	3.35 ± 0.53 ^a^	0.83 ± 0.15 ^a^	0.96 ± 0.10	11.93 ± 0.52 ^a^	0.14 ± 0.06 ^a^	0.49 ± 0.11 ^a^	0.29 ± 0.05 ^a^
HS	0.40 ± 0.03 ^b^	7.48 ± 0.65 ^b^	1.43 ± 0.14 ^b^	1.08 ± 0.09	17.67 ± 0.44 ^b^	1.84 ± 0.13 ^b^	2.03 ± 0.51 ^b^	3.30 ± 1.07 ^b^
HY	0.39 ± 0.02 ^b^	6.12 ± 0.44 ^bc^	1.32 ± 0.20 ^bc^	1.00 ± 0.08	14.67 ± 0.33 ^c^	1.38 ± 0.17 ^b^	1.25 ± 0.21 ^ab^	2.41 ± 0.79 ^b^
HYT5	0.20 ± 0.03 ^a^	3.69 ± 0.53 ^a^	0.87 ± 0.18 ^a^	0.99 ± 0.10	11.87 ± 0.13 ^a^	0.54 ± 0.09 ^a^	0.73 ± 0.09 ^a^	0.49 ± 0.11 ^a^
HYT50	0.21 ± 0.02 ^a^	3.69 ± 0.17 ^a^	0.93 ± 0.11 ^ac^	1.15 ± 0.08	11.67 ± 0.33 ^a^	0.44 ± 0.02 ^a^	0.46 ± 0.06 ^a^	0.39 ± 0.08 ^a^
HYT500	0.22 ± 0.01 ^a^	4.27 ± 0.55 ^ac^	0.89 ± 0.15 ^a^	1.19 ± 0.14	11.83 ± 0.44 ^a^	0.49 ± 0.02 ^a^	0.72 ± 0.24 ^a^	0.71 ± 0.42 ^a^

NS: normal food and saline; HS: HFD and saline; HY: HFD and yogurt; HYT5: HFD and 5 mg/kg of *E. tapos* yogurt; HYT50: HFD and 50 mg/kg of *E. tapos* yogurt; HYT500: HFD and 500 mg/kg of *E. tapos* yogurt; BAT: brown adipose tissue; RpWAT: retroperitoneal white adipose tissue. Values are expressed as mean ± SEM. Different letters indicate a significant difference at *p* < 0.05.

**Table 2:** Liver profile of male and female offspring on PND 21.

Liver Profile of Male Offspring on PND 21									
	Total Protein (g/L)	Albumin (g/L)	Globulin (g/L)	Albumin–Globulin Ratio	ALP (U/L)	AST (U/L)	ALT (U/L)	Gamma-Glutamyl Transferase (U/L)	Total Bilirubin (µmol/L)
NS	56.00 ± 0.40	41.33 ± 1.67 ^a^	18.67 ± 0.33 ^a^	1.77 ± 0.05 ^a^	301.67 ± 9.74 ^a^	152.67 ± 11.67 ^a^	55.33 ± 5.49 ^a^	1.67 ± 0.67 ^a^	2.33 ± 0.33 ^a^
HS	56.50 ± 0.65	35.33 ± 0.33 ^b^	19.33 ± 0.88 ^ab^	2.06 ± 0.03 ^b^	896.33 ± 29.46 ^b^	290.00 ± 7.57 ^b^	102.67 ± 7.42 ^b^	13.00 ± 0.00 ^b^	1.00 ± 0.00 ^b^
HY	57.25 ± 1.11	39.33 ± 0.33 ^ab^	20.00 ± 0.58 ^ab^	1.88 ± 0.01 ^a^	676.00 ± 35.47 ^c^	194.67 ± 6.84 ^a^	85.33 ± 0.88 ^bc^	9.67 ± 3.33 ^bc^	1.00 ± 0.00 ^b^
HYT5	57.25 ± 0.25	41.00 ± 0.58 ^a^	19.33 ± 0.33 ^ab^	1.79 ± 0.02 ^a^	389.00 ± 38.55 ^ad^	161.67 ± 25.83 ^a^	59.67 ± 1.45 ^ad^	1.67 ± 0.33 ^a^	2.33 ± 0.33 ^a^
HYT50	57.75 ± 0.48	38.00 ± 0.58 ^ab^	20.67 ± 0.33 ^ab^	1.85 ± 0.05 ^a^	484.67 ± 53.72 ^d^	178.67 ± 4.67 ^a^	96.33 ± 1.76 ^b^	4.00 ± 1.53 ^ac^	2.33 ± 0.33 ^a^
HYT500	56.50 ± 0.29	40.67 ± 1.45 ^a^	21.67 ± 0.88 ^b^	1.86 ± 0.02 ^a^	528.67 ± 12.45 ^cd^	174.67 ± 15.07 ^a^	74.67 ± 1.86 ^cd^	5.67 ± 1.33 ^abc^	2.33 ± 0.33 ^a^
**Liver Profile of Female Offspring on PND 21**									
	**Total Protein (g/L)**	**Albumin (g/L)**	**Globulin (g/L)**	**Albumin–Globulin Ratio**	**ALT (U/L)**	**AST (U/L)**	**ALP (U/L)**	**Gamma-Glutamyl Transferase (U/L)**	**Total Bilirubin (µmol/L)**
NS	59.17 ± 3.83	41.67 ± 1.45 ^a^	17.00 ± 2.08 ^a^	1.69 ± 0.04 ^a^	246.00 ± 46.65 ^a^	142.00 ± 11.93 ^a^	48.00 ± 5.57 ^a^	1.33 ± 0.33 ^a^	3.00 ± 0.00 ^a^
HS	56.29 ± 1.63	35.33 ± 0.67 ^bc^	23.67 ± 1.86 ^b^	2.04 ± 0.04 ^bc^	633.33 ± 99.09 ^b^	210.00 ± 23.64 ^b^	98.00 ± 2.65 ^b^	13.00 ± 0.00 ^b^	1.33 ± 0.33 ^b^
HY	60.67 ± 1.41	36.67 ± 0.88 ^c^	23.00 ± 0.58 ^b^	1.92 ± 0.04 ^c^	488.00 ± 22.52 ^ab^	191.33 ± 32.42 ^b^	87.00 ± 2.08 ^b^	6.67 ± 3.28 ^ab^	1.33 ± 0.33 ^b^
HYT5	61.00 ± 2.02	41.00 ± 0.58 ^a^	19.33 ± 0.33 ^ab^	1.68 ± 0.03 ^a^	271.00 ± 48.00 ^a^	150.67 ± 9.02 ^a^	47.33 ± 2.19 ^a^	1.33 ± 0.33 ^a^	2.67 ± 0.33 ^ab^
HYT50	59.33 ± 1.23	39.33 ± 0.67 ^ac^	19.67 ± 0.33 ^ab^	1.80 ± 0.07 ^ac^	254.00 ± 30.17 ^a^	148.67 ± 13.09 ^a^	60.33 ± 3.18 ^a^	3.33 ± 1.86 ^a^	2.33 ± 0.33 ^ab^
HYT500	59.29 ± 2.07	38.33 ± 0.33 ^abc^	18.67 ± 0.33 ^ab^	1.75 ± 0.05 ^ac^	305.00 ± 35.03 ^a^	134.33 ± 2.40 ^a^	52.33 ± 6.23 ^a^	3.33 ± 1.86 ^a^	2.33 ± 0.33 ^ab^

NS: normal food and saline; HS: HFD and saline; HY: HFD and yogurt; HYT5: HFD and 5 mg/kg of *E. tapos* yogurt; HYT50: HFD and 50 mg/kg of *E. tapos* yogurt; HYT500: HFD and 500 mg/kg of *E. tapos* yogurt; ALP: alkaline phosphatase; AST: aspartate aminotransferase; ALT: alanine transaminase. Values are expressed as mean ± SEM. Different letters indicate a significant difference at *p* < 0.05.

**Table 3:** Renal profiles of male and female offspring on PND 21.

Renal Profiles of Male Offspring on PND 21					
	Na (mmol/L)	K (mmol/L)	Cl^−^ (mmol/L)	Urea (mmol/L)	Creatinine (mmol/L)
NS	147.33 ± 0.88 ^a^	8.60 ± 0.44 ^ab^	101.67 ± 2.09	6.47 ± 0.15 ^ab^	30.33 ± 0.88 ^a^
HS	141.67 ± 0.33 ^b^	10.03 ± 0.03 ^c^	102.86 ± 2.11	7.53 ± 0.29 ^a^	40.00 ± 1.53 ^b^
HY	143.00 ± 0.58 ^bc^	9.73 ± 0.22 ^ac^	100.67 ± 0.76	5.97 ± 0.03 ^bc^	39.00 ± 1.15 ^bc^
HYT5	145.67 ± 0.33 ^ad^	8.17 ± 0.55 ^b^	100.38 ± 0.78	6.23 ± 0.07 ^ab^	30.67 ± 0.88 ^a^
HYT50	143.67 ± 0.33 ^cd^	8.90 ± 0.21 ^bc^	101.33 ± 1.71	7.27 ± 0.58 ^ab^	34.33 ± 1.45 ^ac^
HYT500	145.67 ± 0.33 ^ad^	8.48 ± 0.13 ^ab^	103.50 ± 1.69	6.07 ± 0.32 ^bc^	32.67 ± 1.20 ^a^
**Renal Profiles of Female Offspring on PND 21**					
	**Na (mmol/L)**	**K (mmol/L)**	**Cl**^**−**^ **(mmol/L)**	**Urea (mmol/L)**	**Creatinine (mmol/L)**
NS	143.83 ± 1.67	8.27 ± 0.27 ^ab^	102.00 ± 1.55	5.57 ± 0.13 ^a^	27.67 ± 4.67 ^a^
HS	144.14 ± 0.83	10.60 ± 0.40 ^c^	103.40 ± 2.18	7.40 ± 0.50 ^b^	39.67 ± 1.67 ^b^
HY	144.00 ± 0.47	9.27 ± 0.27 ^abc^	101.67 ± 0.67	5.93 ± 0.24 ^a^	39.33 ± 0.33 ^b^
HYT5	144.00 ± 0.68	8.03 ± 0.20 ^ab^	102.25 ± 0.25	5.70 ± 0.44 ^a^	28.33 ± 2.03 ^a^
HYT50	142.83 ± 1.25	9.03 ± 0.48 ^ab^	106.00 ± 1.87	6.43 ± 0.09 ^ab^	30.33 ± 2.85 ^a^
HYT500	144.29 ± 0.79	9.53 ± 0.12 ^ac^	104.60 ± 1.03	5.83 ± 0.03 ^a^	28.67 ± 3.18 ^a^

NS: normal food and saline; HS: HFD and saline; HY: HFD and yogurt; HYT5: HFD and 5 mg/kg of *E. tapos* yogurt; HYT50: HFD and 50 mg/kg of *E. tapos* yogurt; HYT500: HFD and 500 mg/kg of *E. tapos* yogurt; Na: sodium; K: potassium; CI^−^: chloride. Values are expressed as mean ± SEM. Different letters indicate a significant difference at *p* < 0.05.

**Table 4:** Lipid profiles of male and female offspring on PND 21.

Lipid Profiles of Male Offspring on PND 21					
	Total Cholesterol (mmol/L)	HDL (mmol/L)	Non-HDL (mmol/L)	LDL (mmol/L)	Triglyceride (mmol/L)
NS	2.13 ± 0.03 ^a^	0.87 ± 0.03 ^a^	0.97 ± 0.03 ^a^	0.73 ± 0.03 ^a^	0.73 ± 0.06 ^a^
HS	2.53 ± 0.03 ^bc^	0.60 ± 0.00 ^b^	1.73 ± 0.03 ^b^	1.30 ± 0.00 ^b^	1.23 ± 0.25 ^b^
HY	2.60 ± 0.06 ^b^	0.53 ± 0.03 ^b^	1.53 ± 0.13 ^b^	1.17 ± 0.07 ^b^	1.15 ± 0.26 ^a^
HYT5	2.27 ± 0.03 ^a^	0.77 ± 0.03 ^a^	1.57 ± 0.07 ^b^	0.90 ± 0.15 ^a^	0.90 ± 0.10 ^a^
HYT50	2.27 ± 0.13 ^a^	0.83 ± 0.03 ^a^	1.63 ± 0.03 ^b^	1.07 ± 0.09 ^b^	1.00 ± 0.26 ^a^
HYT500	2.30 ± 0.00 ^ac^	0.77 ± 0.03 ^a^	1.70 ± 0.06 ^b^	1.13 ± 0.07 ^b^	0.80 ± 0.10 ^a^
**Lipid Profiles of Female Offspring on PND 21**					
	**Total Cholesterol (mmol/L)**	**HDL (mmol/L)**	**Non-HDL (mmol/L)**	**LDL (mmol/L)**	**Triglyceride (mmol/L)**
NS	2.20 ± 0.12 ^a^	0.97 ± 0.03 ^a^	1.60 ± 0.06 ^a^	0.63 ± 0.09 ^a^	0.70 ± 0.10 ^a^
HS	3.00 ± 0.12 ^bc^	0.70 ± 0.00 ^b^	2.10 ± 0.06 ^bc^	1.70 ± 0.06 ^b^	2.13 ± 0.07 ^bc^
HY	2.77 ± 0.12 ^bc^	0.90 ± 0.06 ^a^	2.00 ± 0.10 ^c^	1.43 ± 0.03 ^b^	1.53 ± 0.26 ^c^
HYT5	2.30 ± 0.06 ^ac^	0.90 ± 0.06 ^a^	1.57 ± 0.09 ^a^	1.00 ± 0.06 ^c^	0.97 ± 0.03 ^ac^
HYT50	2.47 ± 0.07 ^ac^	1.00 ± 0.10 ^a^	1.67 ± 0.07 ^ac^	0.90 ± 0.12 ^ac^	1.10 ± 0.15 ^ac^
HYT500	2.17 ± 0.12 ^a^	0.90 ± 0.06 ^a^	1.57 ± 0.09 ^a^	1.07 ± 0.03 ^c^	0.87 ± 0.09 ^a^

NS: normal food and saline; HS: HFD and saline; HY: HFD and yogurt; HYT5: HFD and 5 mg/kg of *E. tapos* yogurt; HYT50: HFD and 50 mg/kg of *E. tapos* yogurt; HYT500: HFD and 500 mg/kg of *E. tapos* yogurt; HDL: high-density lipoprotein; LDL: low-density lipoprotein. Values are expressed as mean ± SEM. Different letters indicate a significant difference at *p* < 0.05.

**Table 5:** Quantitative analysis of fat hypertrophy of male and female offspring on PND 21.

**Quantitative Fat Analysis of Male Offspring on PND 21**		
	**Visceral (µm)**	**RpWAT (µm)**
NS	16.52 ± 1.34 ^a^	16.96 ± 0.98 ^a^
HS	62.37 ± 5.48 ^b^	63.58 ± 3.55 ^b^
HY	36.74 ± 3.30 ^c^	36.84 ± 1.99 ^c^
HYT5	24.58 ± 2.65 ^a^	24.21 ± 2.53 ^a^
HYT50	19.32 ± 0.54 ^a^	19.08 ± 0.47 ^a^
HYT500	21.53 ± 1.29 ^a^	22.05 ± 1.18 ^a^
**Quantitative Fat Analysis of Female Offspring on PND 21**		
	**Visceral (µm)**	**RpWAT (µm)**
NS	16.42 ± 1.55 ^a^	16.16 ± 1.09 ^a^
HS	61.24 ± 5.49 ^b^	62.31 ± 3.45 ^b^
HY	35.83 ± 3.18 ^c^	35.76 ± 1.98 ^c^
HYT5	23.57 ± 2.60 ^a^	23.22 ± 2.56 ^a^
HYT50	20.52 ± 1.28 ^a^	20.40 ± 0.68 ^a^
HYT500	18.27 ± 1.28 ^a^	18.26 ± 1.78 ^a^

NS: normal food and saline; HS: HFD and saline; HY: HFD and yogurt; HYT5: HFD and 5 mg/kg of *E. tapos* yogurt; HYT50: HFD and 50 mg/kg of *E. tapos* yogurt; HYT500: HFD and 500 mg/kg of *E. tapos* yogurt; RpWAT: retroperitoneal white adipose tissue. Values are expressed as mean ± SEM. Different letters indicate a significant difference at *p* < 0.05.

## Data Availability

The datasets generated and/or analyzed during the current study are available from the corresponding author upon reasonable request.
