# Correction: Naomi et al. *Elateriospermum tapos* Yogurt Supplement in Maternal Obese Dams during Pregnancy Modulates the Body Composition of F1 Generation. *Nutrients* 2023, *15*, 1258

**DOI:** 10.3390/nu16091356

**Published:** 2024-04-30

**Authors:** Ruth Naomi, Rusydatul Nabila Mahmad Rusli, Fezah Othman, Santhra Segaran Balan, Azrina Zainal Abidin, Hashim Embong, Soo Huat Teoh, Azmiza Syawani Jasni, Siti Hadizah Jumidil, Khaled Salem Yaslam Ba Matraf, Zainul Amiruddin Zakaria, Hasnah Bahari, Muhammad Dain Yazid

**Affiliations:** 1 Department of Human Anatomy, Faculty of Medicine and Health Sciences, Universiti Putra Malaysia, Serdang 43400, Malaysia; gs60018@student.upm.edu.my (R.N.); rusydatulnabila17@gmail.com (R.N.M.R.); hadizah_jumidil@upm.edu.my (S.H.J.); 2 Department of Biomedical Sciences, Faculty of Medicine and Health Sciences, Universiti Putra Malaysia, Serdang 43400, Malaysia; fezah@upm.edu.my (F.O.); khaled20111268@gmail.com (K.S.Y.B.M.); 3 Department of Diagnostic and Allied Health Sciences, Faculty of Health and Health Sciences, Management and Science University, Shah Alam 40100, Malaysia; santhra@msu.edu.my (S.S.B.); azrina@msu.edu.my (A.Z.A.); 4 Department of Emergency Medicine, Faculty of Medicine, Universiti Kebangsaan Malaysia, Kuala Lumpur 56000, Malaysia; hashimembong77@ukm.edu.my; 5 Advanced Medical and Dental Institute, Universiti Sains Malaysia, Penang 13200, Malaysia; soohuat@usm.my; 6 Department of Medical Microbiology & Parasitology, Faculty of Medicine & Health Science, Universiti Putra Malaysia, Serdang 43400, Malaysia; azmiza@upm.edu.my; 7 Borneo Research on Algesia, Inflammation and Neurodegeneration (BRAIN) Group, Faculty of Medicine and Health Sciences, Sabah Universiti Malaysia, Jalan UMS, Kota Kinabalu 88400, Malaysia; zaz@ums.edu.my; 8 Centre for Tissue Engineering and Regenerative Medicine, Faculty of Medicine, Universiti Kebangsaan Malaysia, Cheras, Kuala Lumpur 56000, Malaysia

## Error in Figure 3 and Legend

After a careful and comprehensive review of our data and the figures in our manuscript, we have identified an area where we believe a correction is warranted in order to enhance the clarity and precision of our findings. Specifically, we wish to replace several micrographs in our published paper [1]. This decision comes after a detailed examination of our datasets and discussions within our team. We have realized that another micrograph from our dataset more accurately represents the treatment group in question and would provide clearer support for our conclusions. This change is not proposed because of any issues with data integrity or duplication, as previously discussed, but rather to ensure that the most representative and precise data are presented. The authors state that the scientific conclusions are unaffected. This correction was approved by the Academic Editor. The original publication has also been updated.

**Figure 3:**
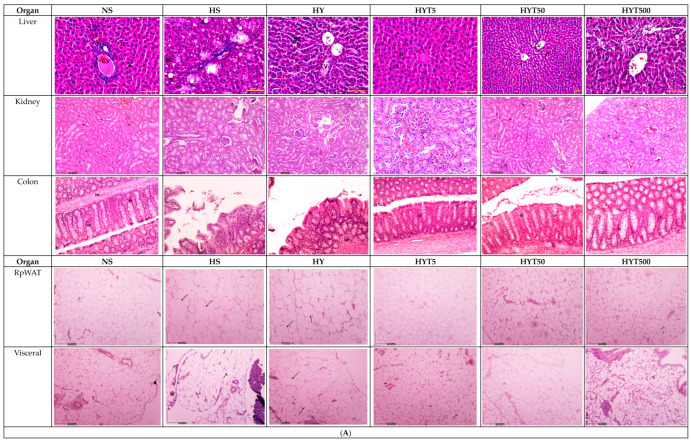

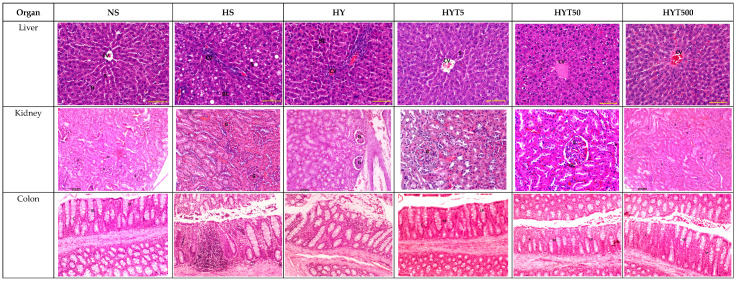

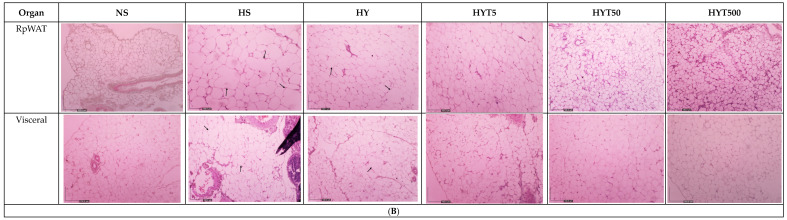
(**A**) The histopathological changes in liver, kidney, colon, RpWAT, and visceral fat tissue of male offspring of PND 21. HS shows abnormal strands of hepato-cytes (H), sinusoids (S), and central veins (CVs). Cell ballooning, steatosis, and >30% of lobular inflammation were seen. The liver histology in the HY group shows abnormal hepatocytes, sinusoids, and CVs with hepatocyte ballooning with lipid droplets. There is no presence of steatosis or lobular inflammation in the HY group. The liver histology for male offspring of the NS, HYT5, HYT50, and HYT500 groups shows a completely normal architecture. The kidneys of male offspring of the HS and HY groups show slight tubular dilation, the presence of abnormal lesions, and slight abnormalities of the renal corpuscle, while the liver histology for male offspring of the NS, HYT5, HYT50, and HYT500 groups shows a completely normal architecture. The colons in male offspring of the HS and HY groups show a detachment of epithelial cells, reduced mucosal content in the colonic wall, severe infiltration in the lamina propria, the presence of inflammation, and fat deposition in the muscle layer. The colon structure of the male offspring in the HYT5, HYT50, and HYT500 groups shows a completely normal architecture. There is severe fat hypertrophy in male offspring of the HS group in retroperitoneal white adipose tissue (RpWAT) and visceral fat tissue. (**B**) The histopathological changes in liver, kidney, colon, RpWAT, and visceral fat tissue of female offspring on PND 21. HS shows abnormal strands of hepatocytes (H), sinusoids (S), and central veins (CVs). Cell ballooning, steatosis, and >30% lobular inflammation were seen. The liver histology in the HY group shows abnormal hepatocytes, S, and CVs with hepatocyte ballooning with lipid droplets. There was no presence of steatosis or lobular inflammation in the HY group. The liver histology for female offspring of the NS, HYT5, HYT50, and HYT500 groups showed a completely normal architecture. The kidneys of female offspring in the HS and HY groups showed slight tubular dilation, the presence of abnormal lesions, and slight abnormalities of the renal corpuscle. The liver histology for female offspring in the NS, HYT5, HYT50, and HYT500 groups showed a completely normal architecture. The colons in female offspring in the HS and HY groups showed a detachment of epithelial cells, reduced mucosal content in the colonic wall, severe infiltration in the lamina propria, the presence of inflammation, and fat deposition in the muscle layer. The colon structure of the female offspring in the HYT5, HYT50, and HYT500 groups shows a completely normal architecture. Severe fat hypertrophy was present in female offspring of the HS group, being observed in retroperitoneal white adipose tissue (RpWAT) and visceral fat tissue. The arrow symbols refer to the fat hypertrophy.
